# Efficacy of octenidine- and chlorhexidine-based wash-mitts against *Candida albicans* and *Candida auris* – a comparative study

**DOI:** 10.1017/ash.2025.82

**Published:** 2025-09-03

**Authors:** Franka Gugsch, Chee Keong Tan, Ding Yuan Oh, Lars Paßvogel, Katrin Steinhauer

**Affiliations:** 1 bactologicum GmbH, Itzehoe, Germany; 2 schülke & Mayr GmbH, Norderstedt, Germany; 3 University of Applied Sciences, Kiel, Germany

## Abstract

**Objectives:** Management of outbreaks of the newly emerging pathogen *Candida auris* may include use of antimicrobial wash-mitts for decolonization. In the absence of large-scale clinical trials, the immediate assessment of the efficacy claims for these products can be based on *in vitro* experimental data that follows the standard protocols established by CEN (European committee for Standardization). In this study, the chemical tolerance of *C. auris* was compared with the surrogate test organism *Candida albicans* as established in the European standards (EN). **Methods:** The study was conducted following the protocol for the quantitative suspension test EN 13624 using *C. albicans* ATCC 10231 in comparison to *C. auris* DSMZ 21092 and *C. auris* DSMZ 105986. Two commercially available wash-mitts containing chlorhexidine digluconate (CHG) or octenidine dihydrochloride (OCT) were used. Experiments were conducted using the impregnation liquid squeezed from the wash-mitts at different dilution concentrations between 0.5% to 97% at a contact time of 30 sec in the presence of 0.03% bovine serum. **Results:** Yeasticidal efficacy according to EN 13624 was found for the OCT wash-mitts at 30 sec at ≥ 10% concentration with *C. albicans* (≥ 4 log RF). In comparison, for both *C. auris* strains ≥ 4 log RF was found at a much lower concentration of ≥ 1%. For the CHG wash-mitts efficacy against *C. albicans* was below 2 log RF at 97% concentration within 30 sec. In contrast efficacy against the two *C. auris* strains was around 3 log RF. **Conclusion:** In conclusion, both *C. auris* strains were found to be significantly more susceptible when compared to *C. albicans* in this study. Moreover, our data also demonstrates that not all antiseptic-impregnated body wipes is equally effective against *C. auris* with OCT having a higher efficacy compared to CHG.

